# The TELEhealth Shared decision-making Coaching and Navigation in Primary carE (TELESCOPE) intervention: a study protocol for delivering shared decision-making for lung cancer screening by patient navigators

**DOI:** 10.21203/rs.3.rs-4254047/v1

**Published:** 2024-04-22

**Authors:** Naomi Q. P. Tan, Lisa M. Lowenstein, Elisa E. Douglas, Jeanne Silva, Joshua M. Bershad, Jinghua An, Sanjay S. Shete, Michael B. Steinberg, Jeanne M. Ferrante, Elizabeth C. Clark, Ana Natale-Pereira, Novneet N. Sahu, Shirin E. Hastings, Richard M. Hoffman, Robert J. Volk, Anita Y. Kinney

**Affiliations:** Rutgers University; The University of Texas MD Anderson Cancer Center; The University of Texas MD Anderson Cancer Center; RWJBarnabas Health Medical Group; Rutgers University; Rutgers Cancer Institute of New Jersey; The University of Texas MD Anderson Cancer Center; Rutgers University; Rutgers University; Rutgers University; Rutgers New Jersey Medical School; Rutgers New Jersey Medical School; Rutgers University; University of Iowa Carver College of Medicine; The University of Texas MD Anderson Cancer Center; Rutgers University

**Keywords:** Shared decision-making, lung cancer screening, lung neoplasms, tobacco treatment, study protocol, cluster randomized controlled trial, telehealth, patient navigation, primary care, enhanced usual care

## Abstract

**Background:**

Lung cancer screening (LCS) can reduce lung cancer mortality but has potential harms for patients. A shared decision-making (SDM) conversation about LCS is required by the Centers for Medicare & Medicaid Services (CMS) for LCS reimbursement. To overcome barriers to SDM in primary care, this protocol describes a telehealth decision coaching intervention for LCS in primary care clinics delivered by patient navigators. The objective of the study is to evaluate the effectiveness of the intervention and its implementation potential, compared with an enhanced usual care (EUC) arm.

**Methods:**

Patients (n = 420) of primary care clinicians (n = 120) are being recruited to a cluster randomized controlled trial. Clinicians are randomly assigned to 1) TELESCOPE intervention: prior to an upcoming non-acute clinic visit, patients participate in a telehealth decision coaching session about LCS delivered by trained patient navigators and nurse navigators place a low-dose CT scan (LDCT) order for each TELESCOPE patient wanting LCS, or 2) EUC: patients receive enhanced usual care from a clinician. Usual care is enhanced by providing clinicians in both arms with access to a Continuing Medical Education (CME) webinar about LCS and an LCS discussion guide. Patients complete surveys at baseline and 1-week after the scheduled clinic visit to assess quality of the SDM process. Re-navigation is attempted with TELESCOPE patients who have not completed the LDCT within 3 months. One month before being due for an annual screening, TELESCOPE patients whose initial LCS showed low-risk findings are randomly assigned to receive a telehealth decision coaching booster session with a navigator or no booster. Electronic health records are abstracted at 6, 12 and 18 months after the initial decision coaching session (TELESCOPE) or clinic visit (EUC) to assess initial and annual LCS uptake, imaging results, follow-up testing for abnormal findings, cancer diagnoses, treatment, and tobacco treatment referrals. This study will evaluate factors that facilitate or interfere with program implementation using mixed methods.

**Discussion:**

We will assess whether a decision coaching and patient navigation intervention can feasibly support high-quality SDM for LCS and guideline-concordant LCS uptake for patients in busy primary care practices serving diverse patient populations.

**Trial Registration:**

This study was registered at ClinicalTrials.gov (NCT05491213) on August 4, 2022.

## BACKGROUND

Lung cancer is the leading cause of cancer death among US men and women, with 21% of all cancer deaths attributed to lung and bronchus cancers. ^1^ When non-small cell lung cancer, the most common type of lung cancer, is detected at the localized stage, the 5-year survival rate is 65%, compared with 37% and 9% at the regional and distant stages respectively. ^2^ Hence, lung cancer screening (LCS) leading to early detection and treatment of lung cancer can reduce lung cancer mortality. Low-dose computed tomography (LDCT) is currently the only screening modality found to reduce lung cancer mortality. ^3^ In 2011, the National Lung Screening Trial (NLST) concluded that screening using LDCT reduced lung cancer mortality by 15–20% compared with using chest radiography. ^3–5^ While LCS using LDCT reduced lung cancer mortality, there were potential harms associated with the screening, including false positive tests, the need for additional testing, possible complications from invasive diagnostic procedures, radiation exposure, overdiagnosis, and incidental findings. ^6^

In 2013, the United States Preventive Services Task Force (USPSTF) issued a Grade B recommendation for LCS using LDCT for individuals age 55–80, who currently smoke or quit smoking within the past 15 years, and who have at least a 30 pack-year smoking history. ^7^ The USPSTF encouraged shared decision-making (SDM) between patients and providers, including a discussion of the potential benefits, harms, and limitations of LCS. ^7^ The 2015 Centers for Medicare & Medicaid (CMS) coverage determination allows for reimbursement of the LDCT for LCS using the same smoking history criteria as the USPSTF but limiting the upper age for screening to 77. ^8^ They also issued an unprecedented coverage requirement that beneficiaries must undergo SDM and counseling delivered by a licensed independent practitioner before screening is offered. ^8^ In 2021, the USPSTF updated their LCS recommendation by lowering the starting age to 50 years and reducing the pack-year threshold to 20. ^9^ These new criteria expanded the pool of eligible persons, particularly high-risk persons of color and women. ^10^ In 2022, CMS updated their coverage determination to align with the new USPSTF criteria for starting age and pack-years. The SDM and counseling visit remains a requirement for CMS reimbursement for the patient’s first LCS, but can now be delivered by a non-clinician and via telehealth. ^11^

LCS uptake has increased slowly since the publication of the NLST main findings, but screening rates remain suboptimal with national estimates of LCS rates among screen-eligible populations ranging from 14.4–21.1%.^12–15^ Clinicians report important barriers to implementing LCS, such as being unfamiliar with screening guidelines, difficulties in identifying eligible patients, lack of training in SDM, lack of time to conduct SDM discussions, competing clinical demands, and need for more support in managing follow-up testing or abnormal results. ^16–18^ Patient barriers include being unaware of the purpose of LCS, not being offered screening, fear of cancer diagnosis, limited access to health care, experiences of smoking-related stigma, and mistrust of the health care system. ^18,19^ These barriers are particularly problematic for underrepresented minorities, people of low socioeconomic status, and rural populations. ^19^ While some experts have attributed the SDM requirement in CMS coverage as being a barrier to screening, ^20,21^ a high quality SDM process has been found to increase patient knowledge about LCS, decrease decisional conflict, increase the likelihood of adherence to screening guidelines, and is aligned with the principle of respecting patient autonomy. ^20,22–24^ Unfortunately, there is evidence that SDM conversations for LCS are not routinely carried out, and many do not meet the minimum criteria for SDM. ^25,26^ Moreover, the potential harms of LCS were infrequently discussed. ^25,26^

To address the deficiencies in SDM, we designed an intervention where decision coaching for LCS is delivered by patient navigators via telehealth to patients recruited from primary care clinics (TELEhealth Shared decision-making COaching and navigation for lung cancer screening in Primary carE [TELESCOPE]). This study builds on our prior work. Our team developed and tested an LCS decision coaching intervention using trained decision coaches and a patient decision aid targeting tobacco quitline callers. ^24,27^ The intervention increased knowledge and improved decision quality. ^27^ This decision coaching intervention is adapted for the TELESCOPE intervention for patients in primary care settings. Delivering decision coaching and navigation via telehealth by patient navigators is a potentially scalable and efficient approach to meeting expectations for delivering SDM and facilitating guideline-concordant screening. In addition, identifying potentially eligible patients from primary care clinics and offering an SDM process can ensure that minority populations are being included in efforts to educate and engage patients in LCS.

### Aims

The trial’s aims are to: (1) test the effectiveness of a decision coaching intervention for LCS delivered by patient navigators (TELESCOPE) versus EUC (enhanced usual care delivered by their primary care provider) on: a) quality of the SDM process about LCS (primary outcome); b) initial LCS uptake and subsequent adherence to annual LCS; and c) tobacco treatment referrals and receipt of tobacco treatment for current smokers; d) assess potential mediators and moderators of intervention effects; and (2) evaluate the implementation potential of patient navigator-delivered decision coaching for LCS by identifying organizational-, clinician-, and patient-level factors that might be barriers or facilitators to successful implementation.

## METHODS

**Figure 1:**
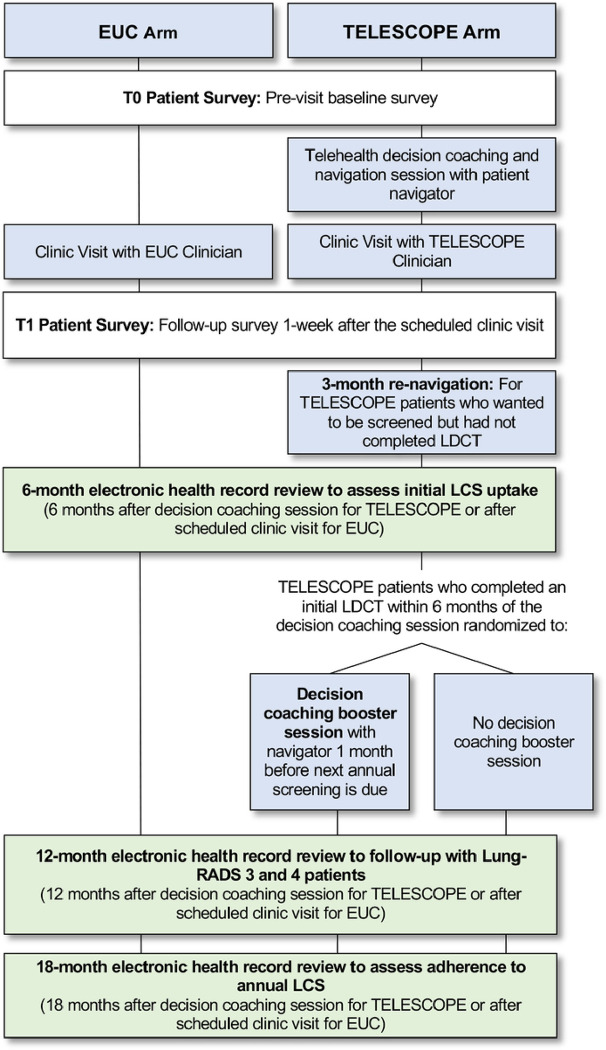
Flow of Patients and Patient Data Collection through the TELESCOPE Trial.

This study protocol is prepared in accordance with The Standard Protocol Items: Recommendations for Interventional Trials (SPIRIT) statement. ^28^ Please see Additional File 1 for the completed SPIRIT checklist and Fig. 1 for the flow of patients and patient data collection for the TELESCOPE trial. The study results will be reported in line with the Consolidated Standards of Reporting Trials (CONSORT) statement for cluster randomized controlled trials (RCTs) ^29,30^ and the implementation outcomes will be reported using the Standards for Reporting Implementation Studies (StaRI) checklist^31^.

### Conceptual Framework

This trial uses an effectiveness-implementation hybrid type I design and is guided by the Practical, Robust Implementation and Sustainability Model (PRISM) as the implementation framework. ^32,33^ PRISM accounts for the role of organizational and patient perspectives, external environment, implementation and sustainability infrastructure, and recipient characteristics in the success of an intervention. ^33^ The impact of the intervention will be evaluated using the constructs in the RE-AIM (reach, efficacy, adoption, implementation, and maintenance) framework, which is a component of PRISM. ^33^ The TELESCOPE intervention uses the implementation strategy of ‘revising professional roles’ by training patient navigators to lead the SDM discussion because clinicians often lack the time and training to provide high quality SDM during clinic visits. ^34^

### Design

**Figure 2:**
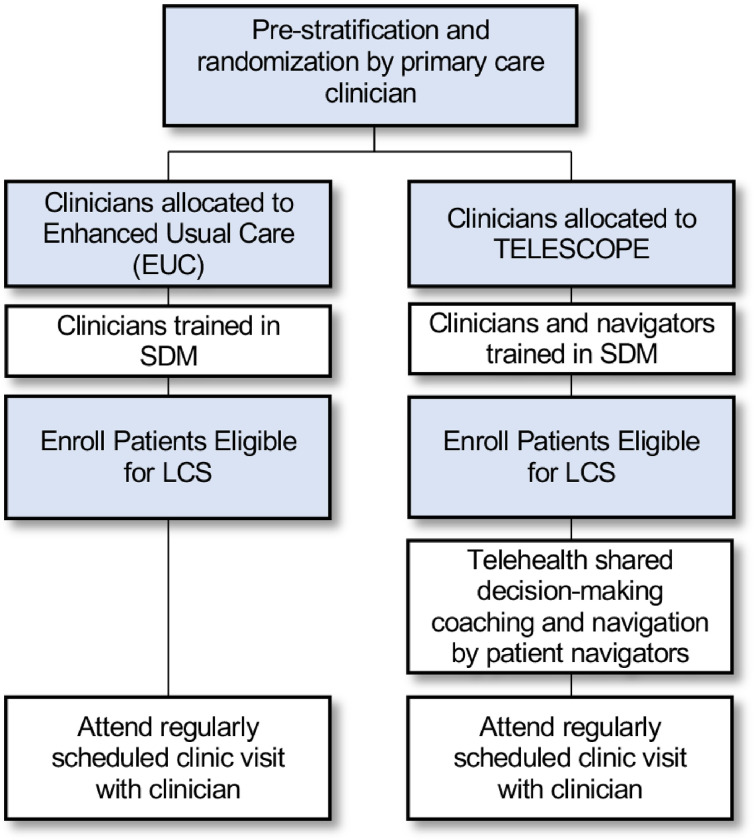
Cluster Randomized Controlled Trial Design for TELESCOPE Trial.

Figure 2 shows an overview of the TELESCOPE study design. We are conducting a cluster RCT with primary care clinicians as the unit of randomization into either (1) the TELESCOPE intervention arm, or (2) an EUC arm. Patients of clinicians in the TELESCOPE arm receive the decision coaching intervention delivered by trained patient navigators (hereafter referred to as ‘decision coaching session’) prior to an upcoming clinic visit with their primary care clinician. Patients of clinicians in the EUC arm have the opportunity to participate in an LCS discussion with their primary care clinician at an upcoming clinic visit. Usual care is enhanced by providing clinicians in both the TELESCOPE and EUC arms with access to a Continuing Medical Education (CME) webinar about LCS and an LCS discussion guide. Patients in the TELESCOPE arm who complete initial screening with negative or benign findings are due for their next screening in 12 months. Half of these participants are randomized to receive a telehealth decision coaching booster session with a patient navigator 1 month before their next annual screening. Table 1 provides a summary of components for the TELESCOPE and EUC arms, which are described in greater detail below.

For the TELESCOPE Trial, a community advisory board of diverse community stakeholders reviews and guides intervention development, recruitment strategies, study procedures, patient-facing materials (e.g., study flyers, informed consent forms, surveys, intervention materials), implementation (e.g., patient recruitment, retention, cultural acceptability), interpretation and dissemination of the results, and identification of next steps.

### Setting and Primary Care Clinicians

The TELESCOPE intervention is implemented remotely through community-based primary care sites affiliated with the RWJBarnabas Health (RWJBH) system, and from academic primary care sites including the Rutgers Robert Wood Johnson Medical School and the Rutgers New Jersey Medical School. RWJBH leadership, academic primary care site leadership, practice administrators, and lead clinicians endorsed involvement of clinicians in the TELESCOPE study. All clinicians (i.e., family medicine physicians, internal medicine physicians, nurse practitioners, physician assistants) from these sites received an initial introduction email from the research team about the TELESCOPE study. For RWJBH clinicians, the study was presented by the clinician administrators at organizational huddles and weekly meetings, and newsletters that described the study were emailed to all clinicians. In line with institutional guidelines, clinicians at academic sites are invited to participate in the study using an opt-out strategy, and non-academic RWJBH clinicians are invited to the study using an opt-in approach. An estimated 120 primary care clinicians (60 in each arm) from 40 statewide primary care sites are participating in the study.

### Patient Eligibility Criteria

Eligibility for this study is based on CMS criteria for LCS to ensure insurance coverage. ^11^ Patients are eligible if they: (1) are aged 50 to 77 years; (2) currently smoke or have quit smoking within the past 15 years; (3) report a 20 or greater pack-year smoking history; (4) are able to speak English; and (5) are scheduled for a non-acute care visit with one of the study clinicians. Individuals are excluded if they have a history of lung cancer, had undergone a chest CT in the past 12 months, have hemoptysis, or their clinician deems them as unable to undergo LCS or subsequent treatment for lung cancer (e.g., being in poor health).

### Patient Recruitment and Enrollment

Using the Epic electronic health record (EHR), study staff identify patients who have an upcoming non-acute clinic appointment with a participating clinician in the next 2 months and who are potentially eligible for the study based on their age and smoking history. Subsequently, participating clinicians are emailed a list of their patients who are potentially eligible and asked to identify and notify study staff within 1 week if any patients on the list should not be contacted to participate in the study. Study staff then send approved potential patient participants a study invitation email containing a link to the pre-screening eligibility form. We also developed a 1-page recruitment flyer with culturally relevant images of patients, key eligibility criteria for the study, study activities, benefits of participating in the study, and study team contact information. The recruitment flyer is sent out to potential patient participants by email (if their email addresses are available), regular mail, and via the Epic patient portal (if their portal is activated). Subsequently, study staff reach out via telephone to potential patient participants to introduce the TELESCOPE study, answer questions, assess interest in participating, determine LCS eligibility, and consent eligible patients who are willing to participate. We employ evidence-based practices for enhancing recruitment and retention including incentives, an interculturally competent recruitment flyer, study specific community advisory board, persistence, skilled teamwork, and an interculturally competent staff. ^35–37^

### Randomization

Participating clinicians are randomized 1:1 to either the TELESCOPE or EUC arm, with stratification based on primary practice affiliation at an RWJBH Primary Care Network or a Rutgers academic primary care practice site, and whether the clinician is a family medicine or internal medicine practitioner. Randomization of clinicians is done by the study’s biostatistician (SS) using STATA. After stratification, randomization is constructed within each stratum with random block sizes ranging from 2 to 6. We cannot blind clinicians or patient participants to the intervention. Patient participants are blinded to the study’s specific hypotheses; statisticians and outcome assessors are blinded to the allocation.

### Description of Intervention

The TELESCOPE intervention involves patient and nurse navigators. The patient navigators (non-clinical) are from the RWJBH Navigation Program and help address patients’ barriers in accessing LCS. Patient navigators receive training (described below) to conduct telehealth LCS education and decision coaching sessions. Nurse navigators supervise patient navigators, place LDCT orders to be signed by clinicians for patients who elect screening, help patients overcome barriers (e.g., cost concerns, transportation, questions about insurance), and navigate patients with cancer or other abnormal findings.

### Intervention Materials

A team of decision scientists and LCS subject matter experts adapted decision coaching materials from a previous decision coaching intervention. ^27^ Adaptation included updating the scientific content to align with extant evidence and CMS guidelines, adding a script for patient navigation (addressing barriers to LCS and identifying screening locations), and adapting the language for primary care settings. The decision coaching materials include: (1) a 2-page **LCS Discussion Guide** with a summary of the benefits and harms of LCS and questions guiding patients in making a shared decision on LCS with their clinician; (2) a **Decision Coaching Manual,** which includes a script for the patient navigator to use during the decision coaching session; (3) **Decision Coaching Slides** which are used by the patient navigator during the session and serve as a visual aid for patients; and (4) **Frequently Asked Questions (FAQs)** to help patient navigators answer patients’ questions.

### TELESCOPE Intervention

Immediately after completing the baseline survey (T0), patients in the TELESCOPE arm are sent the 2-page LCS Discussion Guide and the Decision Coaching Slides via regular mail, and a link to the virtual meeting (Zoom or Microsoft Teams) at their preferred date and time. The decision coaching session is conducted over the phone with patients who do not have internet access. During the decision coaching session, patient navigators follow the scripted Decision Coaching Manual. The script begins with patient navigators introducing themselves and the purpose of the session, and then sharing their computer screen with patients to show the Decision Coaching Slides. Patient navigators review the slides’ content with the patient which includes lung cancer survival rates, importance of yearly LCS, how LCS is performed using an LDCT, potential benefits of LCS (finding lung cancer early and the magnitude of the benefit), and the potential harms of LCS (false alarms, need for additional testing, radiation exposure, overdiagnosis, and incidental findings).

After informing patients of the benefits and harms of LCS, the patient navigator stops sharing their screen and explores the patient’s thoughts about screening, barriers, and screening preferences. Patient navigators are trained in active listening techniques from motivational interviewing to ensure that the patient’s questions, barriers, and concerns are addressed. ^38^ If the patient indicates that she/he wants to be screened, the patient navigator helps identify the patient’s preferred screening location. If patients are unsure or do not want to be screened, the patient navigator advises them to discuss LCS and their concerns with their clinician at their upcoming clinic visit. Finally, the patient navigator assesses patients’ current smoking status, discusses the importance of not smoking, and offers to connect them with a Tobacco Treatment Program.

After the decision coaching session, the patient navigator alerts the nurse navigator to place an order for an LDCT for LCS, typically within 1 week of the patient indicating she/he wants to be screened. The LDCT order is reviewed and signed by the patient’s primary care clinician, typically within 24–48 hours. Nurse navigators follow up with the clinician if the order is not signed during this time period. Study staff reach out to imaging centers within 48 hours of the LDCT order being signed by the clinician to check if the LDCT order has been received and if they have contacted the patient about scheduling. Patients receive a follow-up call from a patient navigator if they have not scheduled their screening within 2 weeks, and a re-navigation call from a patient navigator if they have not completed screening within 3 months.

For TELESCOPE arm patients randomized to receive the decision coaching booster session, patient navigators follow the same process as the initial decision coaching session and a similar script to the Decision Coaching Manual. At the end of the booster session, patients are asked whether they want to pursue screening, and if the answer is yes, similar procedures as abovementioned are followed.

### Navigator Training and Fidelity

All patient navigators receive training on decision coaching, which includes: (1) a **1-hour didactic webinar** delivered on Zoom by two subject matter experts (a decision scientist and a clinician) who cover lung cancer epidemiology, what LCS and an LDCT is, LCS guidelines, the importance of SDM for LCS, and the importance of smoking cessation; (2) a **1-hour role-play session** which includes a 30-minute role-play exercise facilitated by a member of the research team in Zoom breakout rooms where patient navigators take turns using the Decision Coaching Manual with another navigator acting as the patient, followed by a 30-minute review of the role-play exercise by the research team; (3) **2 to 3 practice sessions** using the manual with a mock patient, with recordings of the practice sessions reviewed by the research team; and (4) a **1-hour group review session** on Zoom where the research team presents overall feedback from their review of the patient navigators’ practice session recordings. Once a patient navigator can deliver the decision coaching intervention with good fidelity, she/he starts conducting telehealth decision coaching sessions with patients. Patient navigators who need additional training are asked to conduct 1 to 4 additional practice sessions, reviewed by the research team, until they are able to deliver the script with good fidelity.

Intervention fidelity is evaluated by reviewing the first three decision coaching sessions by each navigator, followed by randomly selected 20% of each navigator’s remaining recorded sessions. We use a pilot-tested fidelity checklist to evaluate whether the scientific information presented is accurate and comprehensive, the navigator is capable of conducting the telehealth session over Zoom/Teams, and the navigator demonstrates good active listening skills. Two reviewers independently complete the fidelity checklist for each recording and conflicts in their ratings are resolved through discussion or adding a third reviewer.

The research team supports patient navigators with ongoing training by: (1) reviewing randomly selected recorded sessions from each patient navigator using the fidelity checklist and providing individual feedback via email; (2) conducting regular review sessions with navigators on Zoom (monthly in the first 6 months, and quarterly until recruitment is completed); (3) sending patient navigators a weekly knowledge check question (e.g., questions about LCS eligibility criteria, screening frequency) or check-in question (e.g., positive experiences or challenging aspects of navigating patients); and (4) conducting refresher training yearly or as needed.

### Enhanced Usual Care (EUC)

Patients in the EUC arm receive care by a clinician who is randomized to this study arm. EUC clinicians are responsible for discussing LCS, addressing the importance of smoking cessation, ordering the LDCT, and following up on the LDCT result with patients.

### Patient Outcomes and Data Collection

After informed consent and enrollment into the trial, all patients complete a baseline survey (T0) and a follow-up survey 1 week after their scheduled clinic visit (T1). Surveys can be completed online, over the phone, or in-person. The study measures and timeline are summarized in Table 2.

### Patient Surveys

The T0 patient survey assesses knowledge of LCS (using the validated LCS-12 measure^39^), decisional conflict (using the validated Decisional Conflict Scale^40^), cancer fatalism^41^, social norms about LCS^42^, medical mistrust^43^, perceived racial discrimination in healthcare^44,45^, health beliefs about LCS^46^, intentions to undergo LCS, and patients’ sociodemographic characteristics, health literacy, and medical history.

The T1 patient survey assesses the study’s primary outcome, the quality of the SDM process, using the SDM Process (SDMP_4) Scale which includes 4 items assessing whether there was a discussion of options, pros, cons, and preferences for LCS. ^47^ Also assessed at the T1 patient survey are knowledge of LCS, decisional conflict, preparation for decision making (using the validated PrepDM scale^48^), description of the LCS discussion with their clinician, LCS health beliefs, intentions to undergo LCS, and satisfaction with telehealth (for TELESCOPE arm patients) ^49^. In line with PRISM constructs (Table 3), the T1 patient survey also assesses TELESCOPE patients’ acceptability of the intervention (using the Ottawa Acceptability Measure^50^).

### Patient Electronic Health Record (EHR) Reviews

A key secondary outcome is initial LCS uptake within 6 months after the decision coaching session (TELESCOPE) or the scheduled clinic visit date (EUC). Other secondary outcomes include adherence to annual LCS among those with an initial low-risk LDCT result by 18 months after the decision coaching session, tobacco treatment referral, receipt of tobacco treatment, and clinical outcomes, including diagnostic testing, cancer diagnoses, and treatment. For the TELESCOPE arm participants, data abstractions are conducted after the initial decision coaching session at 3 months (to trigger re-navigation for those who have not completed screening despite expressing a preference for it), 6 months (to assess initial LCS uptake, tobacco treatment referral/receipt, and clinical outcomes), 12 months (to assess follow-up testing in patients with Lung-RADS^®^ score of 3 or 4, tobacco treatment referral/receipt, and clinical outcomes^51^), and 18 months (to assess adherence to annual LCS, tobacco treatment referral/receipt, and clinical outcomes). For the EUC arm participants, data abstractions are conducted at 6, 12, and 18 months after their initial scheduled clinic visit for the same outcomes.

### Implementation Outcomes and Data Collection

The implementation potential of the TELESCOPE trial is evaluated based on PRISM constructs. ^33^ Table 3 summarizes the data source and measures for each PRISM construct. Surveys are conducted at baseline (T0) for all clinicians, practice administrators, and navigators, and at 12 months (T1) after the first enrolled patient visit for clinicians and navigators. Semi-structured interviews are conducted at T1 for selected clinicians, practice administrators (i.e., medical directors of each site), and navigators. The surveys assess characteristics of organizational recipients and their acceptability of the intervention. The semi-structured interviews assess their perspectives on the intervention, implementation and sustainability infrastructure, and the external environment to identify barriers or facilitators to implementation of the intervention.

### Health Professional Surveys

Clinicians. The T0 clinician survey collects their demographic data, current practices related to LCS, perceptions about organizational readiness for change (ORIC) ^52^, and beliefs about LCS (adapted from LCS-12^39^). The T1 clinician survey evaluates their beliefs about LCS and perceptions about the acceptability of the SDM and counseling visit (using the Acceptability of the Intervention Measure (AIM), Intervention Appropriateness Measure (IAM), and Feasibility of Intervention Measure (FIM) ^53^).

Navigators. The T0 navigator survey collects their demographic data, perceptions about ORIC, and knowledge of LCS (LCS-12^39^). The T1 navigator survey assesses their perceptions about the acceptability of the SDM and counseling visit with the AIM, IAM, FIM measures^53^.

Practice Administrators. The T0 practice administrator survey collects their demographic data and site-level characteristics such as the number of clinicians in their practice, patient volume, and information about clinical workflows related to LCS.

### Health Professional Semi-Structured Interviews

Semi-structured interviews will be conducted with TELESCOPE arm clinicians and navigators (12 months after the initial decision coaching session), EUC arm clinicians (12 months after first EUC patient enrollment), and practice administrators (12 months after the first patient enrollment at their site). To ensure representation of a variety of practice settings and patient populations, clinicians and practice administrators are randomly selected for the interview and are stratified based on the following criteria: internal medicine versus family medicine, academic versus non-academic sites, and RWJBH versus UH sites. We will oversample for clinicians from sites that serve large numbers of Black and Hispanic patients. All navigators involved in the study will be invited to participate in interviews. The semi-structured interview guide is developed based on the PRISM constructs. ^33^ Interviews will take approximately 45 minutes and be audio-recorded.

### Intervention Efficacy and Reach

To assess intervention efficacy, we track the proportion of patients obtaining LCS and tobacco treatment in each study arm. To assess reach of the TELESCOPE intervention, we track the proportion of patients who completed the decision coaching session with the patient navigator among patients consented and randomized to the TELESCOPE arm.

### Sample Size and Power Calculation

Our goal is to randomize approximately 120 primary care clinicians from over 40 primary care practice sites into either the TELESCOPE intervention arm or the EUC arm. We anticipated that it would be feasible to recruit on average 3–4 patients from each of the 120 clinicians, resulting in 420 patients in total (210 patients per arm). We evaluated statistical power and effect sizes based on these assumptions for the quality of the SDM process, our primary outcome, in two-sample *t*-test with adjustment for the intraclass correlation coefficient (ICC) within a practice site and within a clinician. ^54–56^ Assuming 10% attrition by the 1-week follow-up survey, when quality of the SDM process is measured, we would have 189 patients per arm (378 total). Even with the most conservative ICC at 0.20 and Type I error set at 5%, we would have 80% power for a minimal detectable effect size of 0.38. ^57^ Power will be greater for smaller ICCs. Published studies of the SDM Process (SDMP_4) measure have found effects sizes ranging between 0.30 and 0.60. ^58,59^ Hence, a sample of 378 patients by 1-week follow-up provides sufficient power for our primary outcome. We aim to recruit 25% Black and 15% Hispanic patients to ensure the inclusion of these underserved minority populations.

We also evaluated statistical power and effect sizes for LCS uptake at 6 months, our key secondary outcome, in chi-square tests with adjustment for the ICC within a practice site and a clinician. LCS uptake at 6 months is assessed from EHR data; we will use an intention-to-screen analysis to determine the proportion of participants in each arm who completed screening. Assuming an ICC (clinician and practice) of 0.04 and setting the Type I error at 5%, 210 patients per arm yields 80% power to detect a difference in LCS uptake of 14% in the EUC arm compared to 25.5% in the TELESCOPE arm. We will monitor the ICC during data collection and adjust the sample size (i.e., patients) or recruit additional clinicians if the ICC is higher than anticipated.

Power calculations are not needed for the evaluation of TELESCOPE’s implementation potential (Aim 2) as this is primarily descriptive and qualitative in nature. Our target sample size for the surveys is 131, including primary care clinicians, practice administrators, and patient and nurse navigators. We will conduct approximately 41 interviews with selected individuals from these stakeholders across both study arms. We expect that data saturation will be achieved with this amount of interviews. ^60^

### Data Analysis

To assess impact of the intervention on our primary outcome, we will compare the mean difference in quality of the SDM process (i.e., SDMP_4 score) at the 1-week follow-up (T1) between patients in the TELESCOPE arm and EUC arm. Analyses will include two-sample *t*-test adjusted for a clustering effect (at the level of the patients seen by the same clinician), ^61^ as well as linear mixed model (LMM) adjusted for the study design (clustering at the clinician level). We will compare differences in the average percentage of correct responses (LCS knowledge) and mean decisional conflict scores at 1-week follow-up assessment between the two-arms using *t*-test and LMM, adjusting for the study design. We will compare the difference in percentage of patients who smoke and are referred to tobacco treatment services and received tobacco treatment between the two arms using chi-square tests and LMM with logit link, adjusted for the study design. Finally, we will compare the difference in the percentage of LCS uptake between the two arms using chi-square tests and logistic regression, adjusted for the study design. Furthermore, we will also investigate potential moderators [e.g., race and ethnicity, perceived racial discrimination in healthcare, health literacy, smoking history (current vs former smoker, and pack-year smoking history), family vs. internal medicine, academic practice vs, non-academic] using interaction analysis in the regression models. We will document reasons for dropouts, and perform sensitivity analysis with appropriate missing data modeling techniques to inform interpretation of the results. ^62^

For Aim 2, data from the surveys with clinicians, navigators, and practice administrators will be summarized descriptively, including the median, mean, and range, standard deviations, and frequencies. The interview data will be transcribed and analyzed using Template Analysis^63^, which allows for *a priori* and *in-vivo* coding, to examine the culture of the practices as outlined by PRISM. ^33^ The analysis will also examine barriers and facilitators to implementation. Analysis will be carried out by two independent coders and the inter-rater agreement will be calculated. Disagreements in coding will be resolved either by discussion or adding a third coder.

### Data Management

All surveys are hosted on REDCap. To minimize missing data and non-compliance, staff review forms upon receipt to ensure that all items are completed and contact participants to determine if incomplete questions were deliberately skipped and/or to get a response.

### Data Monitoring

To ensure the safety of participants and the integrity of the study, a data safety and monitoring plan is being implemented. As part of this plan, all adverse events are reported to the Rutgers University IRB and the Rutgers Cancer Institute Data Safety Monitoring Board (DSMB). A compiled summary of all reported adverse events is reviewed by the DSMB on a semi-annual basis or more frequently as needed. In addition to the reviews by the DSMB, the principal investigators help ensure the safety of participants, as well as the validity and integrity of the data, by holding weekly meetings with co-investigators and project staff to review study progress and address any issues with the research procedures and database.

### Ethical Considerations

This study is approved by the Institutional Review Board of Rutgers University (also serving as the IRB of record for study activities at MD Anderson Cancer Center). All modifications to the protocol, study procedures, or study materials are submitted to the IRB for approval prior to being implemented. Prior to enrollment in the study, study staff inform potential participants about the study procedures, risks, and benefits. Study staff spend as much time as necessary discussing the consent form with the participants and asking open-ended questions about the consent form to ensure participant comprehension. EHR, survey, interview, and intervention data are stored in a password protected study database on a secure server protected by Rutgers firewall and follows HIPAA regulations. Specific privilege assignments within the study database are limited to the types of functions that authorized users can perform based on their role in the study. Study IDs are assigned for participants to maintain confidentiality. Analyses is limited to the variables necessary for the completion of the proposed study, and results will be reported in aggregate so that individuals are not identifiable.

### Dissemination

The study results will be disseminated through publications in peer-reviewed journals, presentations at professional society meetings and conferences, and will be shared with the TELESCOPE community advisory board members and study participants. The findings will also be disseminated to the study’s health care settings and New Jersey residents, facilitated by Rutgers Cancer Institute’s community outreach and engagement team.

## DISCUSSION

The goal of the TELESCOPE trial is to test a telehealth decision coaching intervention for LCS that can be implemented in real-world primary care settings serving racially, ethnically, and socioeconomically diverse patient populations. The TELESCOPE intervention involves patient navigators delivering a telehealth decision coaching session about LCS to screen-eligible patients prior to a primary care clinician visit. This novel approach has the potential to advance the implementation of SDM and improve guideline-concordant uptake of LCS by providing patients with high-quality decision support about LCS and decreasing the burden on busy primary care clinicians.

A strength of the trial is the recruitment of patient participants from a large integrated health system comprising a network of primary care sites across the state, including both community-based and academic sites, and institutional buy-in and collaboration from these sites. There is a growing trend of healthcare consolidation and it is estimated that 49% of primary care physicians and 72% of hospitals are affiliated with a large health system. ^64^ Hence, the results of this study may inform the development of scalable LCS interventions in other integrated health care systems. In addition, the mix of recruitment sites increases access to underserved minority and low-income participants, who have been underrepresented in LCS interventions thus far. Furthermore, the addition of a decision coaching booster session, conducted before the next annual screening is due for randomly selected patients, may improve adherence to annual LCS, which is crucial for reducing lung cancer mortality.

### Trial Status

Recruitment started in May 2023. As of early April 2024, 117 clinicians were participating in the trial and 134 patient participants have enrolled in the study. Recruitment of participants is expected to be completed by May 2026.

## Figures and Tables

**Table 1:** Summary of the Study Components for the TELESCOPE and Enhanced Usual Care (EUC) Arms.

Participants	TELESCOPE Arm	EUC Arm
Clinicians	• Provided with access to CME webinar on lung cancer screening (LCS) and shared decision-making (SDM) • 2-page LCS Discussion Guide summarizing LCS eligibility, benefits/harms of LCS to consider, insurance coverage, and screening decisions	Same as TELESCOPE arm
Navigators	Navigator Pre-Intervention Training: • 1-hour live didactic webinar on LCS and SDM • 30-minute role play session using the decision coaching materials^a^ and 30-minute review of the role play session • 2–3 practice decision coaching sessions with mock patients • 1-hour group review of the practice sessions Navigator Ongoing Training: • Monthly review meetings with navigators in first 6 months, then quarterly meetings until recruitment is completed • Weekly knowledge check question • Yearly refresher training Intervention Fidelity: • Review of first 3 recorded coaching sessions using a fidelity checklist and thereafter randomly selected recordings; with individual feedback provided to each patient navigator	Not applicable (no EUC navigators)
Patients	Telehealth Decision Coaching Session: • Conducted on Zoom/Teams by patient navigator using decision coaching slides • Navigators provide information about the LDCT procedure, benefits and harms of LCS • Navigator assesses and address patient barriers • Navigator assesses screening preferences and places LDCT order if patient elects screening • Navigator offers referral for tobacco treatment to patients who are interested Follow-Up Navigation: • 2 weeks after order is placed and signed: Navigator follows up with patients who wanted to be screened but did not schedule the LDCT yet • 3 months after initial decision coaching session: Navigator re-navigates patients who wanted to be screened but did not complete LCS • Decision coaching booster session: 50% of patients who completed an initial LCS and have low-risk findings are randomly selected to receive a decision coaching booster session 1 month before their next annual screening is due	Clinic visit with EUC arm clinician: EUC clinicians are responsible for discussing LCS, addressing smoking cessation, ordering the LDCT, and following up on the LDCT result with patients.

a The decision coaching materials include: (1) a 2-page LCS Discussion Guide with a summary of the benefits and harms of LCS and questions guiding patients in making a shared decision on LCS with their clinician; (2) a Decision Coaching Manual, which includes a script for the patient navigator to use during the decision coaching session; (3) Decision Coaching Slides which are used by the patient navigator during the session and serve as a visual aid for patients; and (4) Frequently Asked Questions (FAQs) to help patient navigators answer patients’ questions.

**Table 2:** Patient-Reported Measures and Timepoints.

Construct/Measures	No. of Items	Baseline Survey (T0)	1-Week Survey (T1)^a^	6-Month EHR Review^b^	12-Month EHR Review^b^	18-Month EHR Review^b^
Sociodemographics, Health Literacy, Medical History^65,66^	19	X				
Quality of SDM Process^47^	4		X			
Knowledge of LCS^39^	12	X	X			
Decisional Conflict^40^	10	X	X			
Preparation for Decision Making (PrepDM) ^48^	10		X			
LCS Discussion with Clinician	1		X			
Cancer Fatalism^41^	3	X				
Social Norms^42^	6	X				
Medical Mistrust^43^	7	X				
Perceived Racial Discrimination in Healthcare^44,45^	2	X				
LCS Health Beliefs^46^	35	X	X			
Intention to Undergo LCS	2	X	X			
Telehealth Patient Satisfaction^49^	6		X			
Ottawa Acceptability measure^50^	10		X			
Initial LCS Uptake	-			X		
Follow-Up on Lung-RADS 3 or 4 cases	-				X	
Adherence to Annual LCS	-					X
Tobacco Treatment Referral	-			X	X	X
Receipt of Tobacco Treatment	-			X	X	X
Cancer Diagnoses and Treatment	-			X	X	X

a The T1 survey is administered 1-week after the scheduled clinic visit for both TELESCOPE and EUC arm patients.

b The 6-, 12-, and 18-month data abstractions are from the initial decision coaching session for TELESCOPE arm patients and from the scheduled clinic visit for EUC arm patients.

**Table 3:** Implementation Measures based on Practical, Robust Implementation and Sustainability Model (PRISM) Constructs.

PRISM Construct Assessed	Data Source and Timing	Measure(s)
Reach	Ongoing Electronic Health Record (EHR) review	Proportion of patients who completed the decision coaching session with the patient navigator among patients consented and randomized to the TELESCOPE arm
Implementation (Fidelity)	Recordings of first 3 decision coaching sessions of each patient navigator, then a random 20% of each patient navigator’s remaining sessions	Fidelity checklist to evaluate patient navigators’ 1) telehealth technical skills (4 items), 2) content of SDM conversations (16 items), and 3) decision coaching skills (6 items)
Characteristics of the Recipients – Organizational Perspective	T0^a^ practice administrators survey	Site-level characteristics questions
	T0 and T1^a^ clinician surveys	Demographic questions; Organizational Readiness for Change (ORIC) measure^52^; Beliefs about LCS (adapted from LCS-12) ^39^
	T0 navigator survey	Organizational Readiness for Change (ORIC) measure^52^
Intervention Characteristics – Organizational Perspective	T1 clinician survey, T1 navigator survey	Acceptability of the intervention measure (AIM), intervention appropriateness measure (IAM), and feasibility of intervention measure (FIM) ^53^
Intervention Characteristics – Patient Perspective	T1 patient survey (1 week follow-up survey after the scheduled clinic visit)	Ottawa Acceptability Measure^50^
Intervention – Organizational Perspective; Characteristics of Recipients – Organizational Perspective; Implementation and Sustainability Infrastructure; External Environment	Semi-structured interviews with clinicians, practice administrators, navigators^b^	Semi-structured interview guide will reflect PRISM constructs
Efficacy (Patients)	Ongoing EHR review	Proportion of patients obtaining lung cancer screening and tobacco treatment in each study arm

a T0 surveys for all health professionals take place prior to enrollment of patients into the study. T1 surveys for clinicians and navigators for take place 12 months after the first enrolled patient visit. Practice administrators do not complete T1 surveys.

b Semi-structured interviews with health professionals take place 12 months after the initial decision coaching session for TELESCOPE arm clinicians and navigators, and 12 months after the first patient enrollment at their site for EUC clinicians and practice administrators.

## Data Availability

The intervention materials are available upon request from the MD Anderson Cancer Center Decision Science Core Facility (decisionscience@mdanderson.org).

## List Of Abbreviations

LCS: Lung Cancer Screening
LDCT: Low-Dose Computed Tomography
NLST: National Lung Screening Trial
USPSTF: United States Preventive Services Task Force
SDM: Shared Decision-Making
CMS: Centers for Medicare & Medicaid Services
TELESCOPE: TELEhealth Shared decision-making COaching and navigation in Primary carE
EUC: Enhanced Usual Care
SPIRIT: Standard Protocol Items:Recommendations for Interventional Trials
CONSORT: Consolidated Standards of Reporting Trials
StaRI: Standards for Reporting Implementation Studies
RWJBH: RWJBarnabas Health
EHR: Electronic Health Record
CME: Continuing Medical Education
ICC: Intraclass Correlation Coefficient
LMM: Linear Mixed Model
DSMB: Data Safety Monitoring Board
